# Early Endoscopic Features of Suspected Autoimmune Gastritis in a Young Adult Male Patient: A Case Report

**DOI:** 10.7759/cureus.115319

**Published:** 2026-08-27

**Authors:** Ichiro Imoto, Toshio Katoh, Satoko Oka, Kae Konishi, Sawa Nakauchi, Noriyuki Horiki, Esteban C Gabazza

**Affiliations:** 1 Digestive Endoscopy Center, Doshinkai Tohyama Hospital, Tsu, JPN; 2 Department of Surgery, Doshinkai Tohyama Hospital, Tsu, JPN; 3 Department of Internal Medicine, Doshinkai Tohyama Hospital, Tsu, JPN; 4 Digestive Disease Center, Mie University Faculty and Graduate School of Medicine, Tsu, JPN; 5 Department of Immunology, Mie University Faculty and Graduate School of Medicine, Tsu, JPN

**Keywords:** antiparietal cell antibody, autoimmune gastritis, corpus-predominant atrophy, early stage, type a gastritis

## Abstract

Autoimmune gastritis, traditionally referred to as type A gastritis, is characterized by corpus-predominant atrophic gastritis caused by autoimmune mechanisms. Most cases are diagnosed in middle-aged or elderly individuals, as complications such as pernicious anemia and impaired absorption of iron and vitamin B12 typically manifest in advanced stages. Additionally, patients with autoimmune gastritis are often asymptomatic, making reports of early-stage endoscopic findings exceedingly rare. A 22-year-old male presented to our hospital with complaints of epigastric pain and lower back pain. He had undergone eradication therapy for *Helicobacter pylori *infection at another hospital three months prior to presentation. A urea breath test confirmed successful eradication of *H. pylori*. Endoscopic examination revealed extensive, sharply demarcated mucosal atrophy extending orally from the middle of the gastric body, while the gastric antrum showed no evidence of atrophy or intestinal metaplasia. Laboratory tests revealed a mild elevation in anti-parietal cell antibody levels, with a titer of 1:10, whereas serum gastrin and vitamin B12 levels remained within normal limits. Iron metabolism parameters were also normal. However, histopathological confirmation was unavailable, and thus a definitive diagnosis of autoimmune gastritis could not be established. This report presents a rare case of suspected early-stage autoimmune gastritis with distinctive endoscopic findings in a young male.

## Introduction

Atrophic gastritis, an established risk factor for gastric cancer, is most commonly associated with *Helicobacter pylori* infection [1]. In *H. pylori*-associated atrophic gastritis, inflammation and atrophy typically develop in the antrum and incisura and may subsequently extend proximally, producing multifocal atrophic changes. By contrast, autoimmune gastritis (AIG), historically referred to as type A gastritis, is characterized by immune-mediated destruction of parietal cells in the oxyntic mucosa, resulting in corpus- and fundus-predominant inflammation and atrophy with relative sparing of the antrum [2]. As AIG progresses, patients may develop iron deficiency, vitamin B12 deficiency, pernicious anemia, and neurological manifestations [3]. Nevertheless, many patients remain asymptomatic, and the condition may be detected incidentally during endoscopic or laboratory evaluation.

The diagnosis of AIG is based on a combination of clinical, endoscopic, histopathological, and serological findings, although universally accepted diagnostic criteria have not been established. In 2023, the Study Group on Establishment of Diagnostic Criteria for Type A Gastritis, an adjunctive group of the Japanese Society of Gastrointestinal Endoscopy, proposed diagnostic criteria for AIG [4]. These criteria describe characteristic endoscopic and histopathological findings and include gastric autoantibodies, such as anti-parietal cell and anti-intrinsic factor antibodies. Potential AIG generally refers to the presence of gastric autoantibodies without corresponding endoscopic or histopathological abnormalities. By contrast, early-stage AIG is characterized by emerging corpus-predominant abnormalities before the development of advanced mucosal atrophy or clinically evident consequences such as hypergastrinemia, iron deficiency, and vitamin B12 deficiency. Under the Japanese criteria, however, confirmation of early-stage AIG requires characteristic histopathological findings.

Although interest in the early endoscopic features of AIG has increased, reports describing such findings in young adults remain limited [5-7]. Here, we describe a 22-year-old man with corpus-predominant endoscopic abnormalities suggestive of early-stage AIG and weakly positive anti-parietal cell antibodies, but without anemia, iron deficiency, vitamin B12 deficiency, or hypergastrinemia. Because histopathological confirmation was not obtained, this case is presented as suspected rather than confirmed early-stage AIG. This article was previously posted to the Preprints.org preprint server (https://www.preprints.org/manuscript/202501.1950) on January 26, 2025.

## Case presentation

A 22-year-old man presented with epigastric pain and lower back pain. His medical history was unremarkable except for successful *H. pylori *eradication therapy performed at another hospital three months before presentation.

Laboratory investigations revealed no evidence of anemia, iron deficiency, or vitamin B12 deficiency, and serum gastrin was within the reference range. Anti-parietal cell antibodies were weakly positive at a titer of 1:10 by indirect immunofluorescence assay (Fujirebio Inc., Tokyo, Japan) (Table 1).

**Table 1:** Clinical parameters of the patient

Parameter	Results	Reference range	Unit
White blood cell count	4.1	3.3-8.6	1000/μL
Neutrophils	52.0	36.0-69.5	%
Lymphocytes	35.2	20.8-52.7	%
Monocytes	5.9	4.5-10.6	%
Eosinophils	3.9	0.5-8.6	%
Basophils	0.8	0.2-1.7	%
Red blood cell count	5.2	4.35-5.55	million/μL
Hemoglobin	17.4	13.7-16.8	g/dL
Hematocrit	49.8	40.7-50.1	%
Mean corpuscular volume	95.9	86.6-98.2	fL
Mean corpuscular hemoglobin	33.5	27.5-33.2	pg
Mean corpuscular hemoglobin concentration	35.0	31.7-35.3	g/dL
Platelet count	254.0	158-348	1000/μL
Serum iron	161.0	40-188	μg/dL
Total iron-binding capacity	336.0	253-365	μg/dL
Transferrin saturation	47.9	20-50	%
Serum ferritin	58.2	31-325	ng/mL
Anti-parietal cell antibody	10.0	0-9	-
Serum gastrin	170.0	0-200	pg/mL
Serum vitamin B12	497.0	180-914	pg/mL
Fasting blood glucose	85.0	70-109	mg/dL
Total protein	7.5	6.6-8.1	g/dL
Albumin	4.9	4.1-5.1	g/dL
Albumin-to-globulin ratio	1.9	1.32-2.23	-
Total bilirubin	1.1	0.4-1.5	mg/dL
Aspartate aminotransferase (glutamic-oxaloacetic transaminase)	18.0	13-30	U/L
Alanine aminotransferase (glutamic-pyruvic transaminase)	21.0	10-42	U/L
Blood urea nitrogen	19.0	8.0-20.0	mg/dL
Serum creatinine	0.9	0.85-1.07	mg/dL
Estimated glomerular filtration rate	95.4	≥60	mL/min
*Helicobacter pylori* urea breath test	1.1	0-2.4	‰ (per mille)

**Figure 1:**
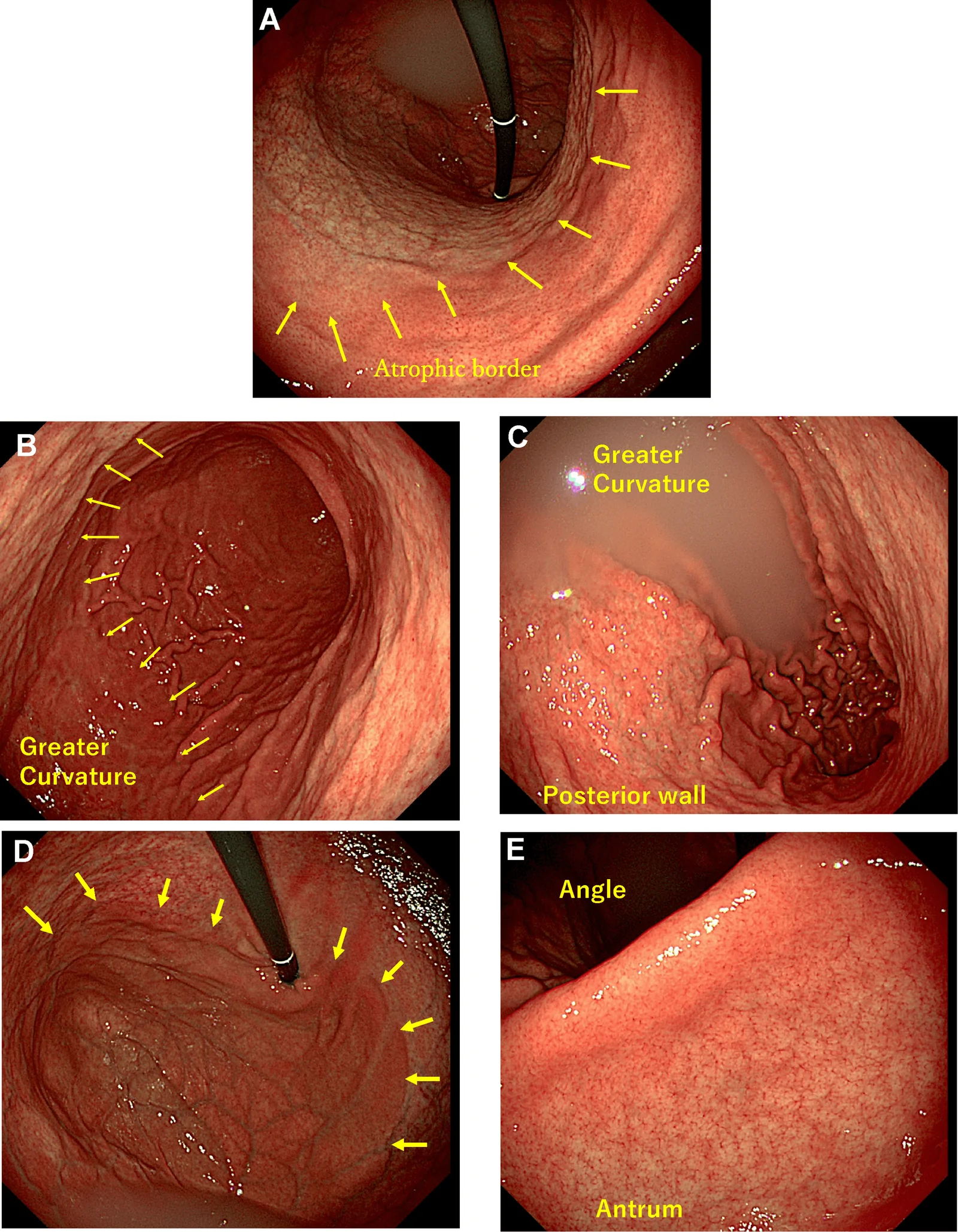
Gastroscopic early findings of autoimmune gastritis (A) The lesser curvature of the lower gastric body shows a regular arrangement of collecting venules with a sharply demarcated atrophic border between the lower and middle corpus. (B) The greater curvature of the lower body appears non-atrophic, whereas (C) the middle and upper segments exhibit extensive atrophy. (D) The upper body and fundus display scattered diffuse redness and atrophy. (E) The antral mucosa shows transvascular findings without severe atrophy or intestinal metaplasia.

Successful eradication of *H. pylori* was confirmed by a urea breath test. Upper gastrointestinal endoscopy was subsequently performed using an Olympus GIF-290 endoscope. In the lower gastric body, an upward view of the lesser curvature demonstrated a regular arrangement of collecting venules (RAC), an endoscopic feature consistent with *H. pylori*-negative gastric mucosa [8]. A well-defined atrophic border was identified between the lower and middle gastric body (Figure 1A); a downward view of the greater curvature showed preserved mucosa in the lower corpus (Figure 1B), whereas the middle and upper corpus exhibited diffuse mucosal atrophy (Figure 1C). Scattered diffuse redness was observed in the upper corpus and fundus (Figure 1D). In contrast, the antral mucosa was well preserved, with visible submucosal vessels and no evidence of significant atrophy or intestinal metaplasia (Figure 1E).

Taken together, corpus-predominant atrophy with relative antral preservation, weak anti-parietal cell antibody (APCA) positivity, and normal vitamin B12, iron, and gastrin levels suggested the possibility of early-stage AIG, before the development of significant functional impairment. Histopathological confirmation could not be obtained because the patient resided in another region and was unable to return to our institution for biopsy.

## Discussion

AIG is generally considered a slowly progressive disease that evolves through several pathological stages. Initially, circulating anti-parietal cell and/or anti-intrinsic factor antibodies may be detected in the absence of histological or endoscopic abnormalities, a phase referred to as potential AIG [9]. Histological inflammation of the corpus subsequently develops before endoscopic atrophy becomes apparent. In the Japanese diagnostic criteria for AIG, endoscopic features of early-stage disease were not included because further accumulation and characterization of clinical endoscopic data were considered necessary. Therefore, the diagnosis of early-stage AIG requires only histological confirmation and a positive gastric autoantibody test [4].

As the disease progresses, corpus-predominant atrophy becomes evident on endoscopy, although many patients remain asymptomatic. In advanced disease, progressive destruction of parietal cells leads to hypochlorhydria, hypergastrinemia, iron deficiency, vitamin B12 deficiency, and ultimately to pernicious anemia [10].

The present patient appeared to represent an early stage of this disease spectrum. Despite characteristic corpus-predominant atrophic changes on endoscopy and weakly positive APCA, the levels of serum gastrin, vitamin B12, and iron parameters remained within the normal range. The absence of anemia or nutritional deficiencies further supports the likelihood that the disease had been identified before progression to advanced AIG.

Recognition of early AIG remains challenging because most patients are asymptomatic and undergo endoscopy only for unrelated indications [10-11]. Consequently, the endoscopic characteristics of early disease have been described in only a limited number of studies. Kotera et al. reported longitudinal pseudopolyps and erythematous, swollen mucosa as characteristic findings in 12 patients with early AIG, with a mean age of 56.2 years (range, 41-71 years) [7]. In contrast, our patient was only 22 years old and did not exhibit these characteristic findings, although scattered diffuse redness was seen in the upper corpus and fundus. Instead, the most striking feature was a sharply demarcated, corpus-predominant atrophic border with preservation of the antral mucosa, a pattern that suggested AIG rather than *H. pylori*-associated gastritis, despite a history of successful *H. pylori* eradication. Generally, in *H. pylori* infection, atrophy on the greater curvature of the gastric body progresses from the lower to the upper portion [12]; however, in this case, atrophy was more advanced in the middle to upper portions than in the lower portion (Figures 1B-1C). This suggests that the atrophy in this case was most likely caused by AIG.

The increasing recognition of AIG following successful *H. pylori* eradication has important clinical implications. In countries such as Japan, where endoscopic screening is widely performed and *H. pylori* eradication is common [13], corpus-predominant atrophy without corresponding antral involvement should prompt consideration of AIG. Furthermore, previous studies have suggested that achlorhydria associated with AIG may contribute to false-positive urea breath tests because urease-producing non-*H. pylori* bacteria can colonize the stomach. Awareness of this phenomenon may facilitate appropriate interpretation of diagnostic tests in selected patients [14].

This case has several limitations. First, histopathological confirmation could not be obtained because the patient lived far from our institution and was unable to attend the scheduled follow-up examination. According to the Japanese diagnostic criteria, early-stage AIG requires histopathological confirmation. Therefore, this case did not meet the diagnostic criteria for early-stage AIG and should be considered suspected rather than confirmed AIG. Second, anti-intrinsic factor antibodies were not measured because testing is not covered by the Japanese national health insurance system and would have required a costly out-of-pocket examination. The clinical suspicion of AIG was based primarily on the endoscopic findings, weak APCA positivity, and the absence of evidence of persistent *H. pylori* infection. However, the weakly positive APCA at a low titer of 1:10 should be interpreted with caution, as it may represent a false positive and is insufficient on its own to confirm AIG.

Despite the absence of histopathological confirmation and anti-intrinsic factor antibody testing, the overall clinical presentation was considered consistent with early-stage AIG according to the recently proposed Japanese diagnostic criteria [4]. Endoscopic findings may be absent or subtle in pediatric AIG [15], and our literature review identified no studies clearly establishing when characteristic endoscopic changes first emerge during disease progression. Although histopathological evaluation remains an important component of the diagnosis of AIG, enterochromaffin-like cell hyperplasia may be absent during the early stage [4]. Furthermore, inflammation and parietal cell loss are not specific to AIG and may also occur in *H. pylori*-associated gastritis, making histopathological diagnosis challenging in the early phase of the disease [16]. Therefore, careful recognition of characteristic endoscopic findings may play a particularly important role in identifying early-stage AIG [4]. In this context, the present case provides potentially valuable information regarding the endoscopic features of AIG at an early stage.

## Conclusions

In conclusion, this case describes a young adult with endoscopic findings suggestive of early-stage AIG in the absence of hypergastrinemia, vitamin B12 deficiency, iron deficiency, or anemia. Although bamboo-like and salmon roe-like appearances were absent, endoscopy revealed a distinct atrophic border between the pyloric and fundic gland regions, corpus-predominant atrophy, and relative antral preservation. However, this distribution is not pathognomonic for AIG. Given the weak APCA positivity, lack of anti-intrinsic factor antibody testing, and absence of histopathological confirmation, alternative causes could not be completely excluded. Therefore, this case should be regarded as suspected rather than confirmed early-stage AIG. Recognition of these subtle endoscopic features may prompt further diagnostic evaluation, including histopathological assessment, and appropriate clinical follow-up.
